# Healthcare waste in German hospitals: A nationwide benchmark study

**DOI:** 10.1177/0734242X251405987

**Published:** 2026-01-13

**Authors:** Anton Vielsack, Viola Galler, Anne-Kathrin Cassier-Woidasky, Franziska Zecha, Joerg Woidasky

**Affiliations:** 1 University of Applied Sciences Pforzheim, Pforzheim, Germany; 2 University of Trier, Trier, Germany; 3 htw saar, Saarbrücken, Germany

**Keywords:** Infectious waste, hospital waste, healthcare waste, clinical waste, circular economy, Germany

## Abstract

There is currently no publicly available, scientifically sound data on solid waste generation in German hospitals, which is one reason why the implementation of circular economy initiatives on this subject has so far been largely unsuccessful. Therefore, this study aims to conduct a nationwide field study on waste generation in German hospitals. To this end, waste and structural data from 122 German hospital locations covering the 4 clusters of general hospitals, university hospitals, specialist hospitals and other hospitals in all 16 German states were collected in 2024 and statistically analysed. A total of 103,432 Mg of waste from these hospitals was documented, of which around 9% was hazardous and 1.4% infectious. The results show strong correlations between waste generation and staffing levels. Furthermore, differences between general and university hospitals were found for all eight key performance indicators examined. Based on the results, a differentiated extrapolation model for the mass of waste from German hospitals, which uses the number of beds as a reference value, is proposed.

## Introduction

Increasing waste generation in the healthcare sector not only leads to an increasing burden on healthcare facilities, but also intensifies the negative impact on the environment (Karliner et al., 2019; WHO, 2022). According to the German Federal Statistical Office (2022), around 425,400 Mg of waste that is specifically attributable to the healthcare sector (waste group 18 01) is generated in Germany annually. Of this, 2.4% is infectious waste (10,100 Mg/a; waste code 18 01 03*). To date, almost all hospital-specific waste in Germany is incinerated. Although thermal energy can be recovered depending on the type of plant and the hazardousness of the waste, material recycling for non-infectious solid hospital waste would already be possible today under certain conditions relating to health and safety (LAGA, 2021).

Both the current literature (Mol et al., 2022; Singh et al., 2022; Slutzman et al., 2023) and the analysis of official statistics at federal (Federal Statistical Office, 2022) and state levels (Federal Statistical Office, 2008, 2024a) indicate substantial data deficiencies with regard to the detailed knowledge on waste generation in German hospitals, in particular to the infectious waste type 18 01 03*. A substantial lack of scientific publications on waste generation in the healthcare sector in Germany can be stated, although the analysis of waste from hospitals along with the number of journal articles on this subject has risen sharply worldwide since 2000 (Singh et al., 2022).

As shown in a related, unpublished work (Vielsack et al., manuscript currently under review), data currently publicly available are insufficient to enable a detailed, management-oriented analysis of waste generation at the hospital level in Germany, as the available aggregated data on hospital waste lack granularity and are missing relevant hospital-specific structural information (Diaz et al., 2008; Feld et al., 2023; Hunger et al., 2023; Mol et al., 2022). To address this, a field study was conducted to generate scientifically robust and reliable data on hospital waste, including hospital-specific structural information. These data are essential not only to close a major knowledge gap, but also to support evidence-based decision-making, optimize hospital solid waste management and provide a foundation for future circular economy initiatives in the healthcare sector.

In Germany, healthcare waste is classified according to the European Waste Catalogue and the Waste Catalogue Ordinance (Abfallverzeichnisverordnung – AVV). This study focuses on AVV group 18 01, which covers human healthcare waste. The types most relevant here are sharps (18 01 01), infectious waste (18 01 03*) and non-infectious waste (18 01 04), while the other types mainly consist of chemical or pharmaceutical wastes. In addition, healthcare facilities generate other wastes such as paper and cardboard (waste codes 15 01 01, 20 01 01) or mixed municipal waste (20 03 01). Handling and disposal follow the guidance document ‘LAGA M18’ issued by intergovernmental, ‘working group on waste of federal states and the federation’ (Bund-/Länder-Arbeitsgemeinschaft Abfall – LAGA).

The overall objective of this study is to quantify hospital-level waste generation and structural data across Germany, and to integrate these datasets to provide a scientifically robust basis for evidence-based hospital waste management and future circular economy strategies in health care.

## Materials and methods

### Data collection and structure

All German acute hospitals were contacted via the state hospital associations in an e-mail campaign, informing about the online survey. In some cases, both the state hospital associations and individual participating hospitals were contacted primarily by telephone to ask about forwarding or participation. The online survey with the (online) survey tool LimeSurvey was open from 20 July 2024 to 5 December 2024. The online questionnaire used comprised five parts: Part A asked for contact details, communication channels and consent. In Part B, the waste masses (in Mg) for 55 waste code numbers for the calendar year 2023 were collected, focusing on waste group 18 01. In Part C, 17 hospitals’ structural information was collected for the year 2023 (reference date 31 December 2023). In Part D, the specialist services provided by the hospital was asked for (reference date 31 December 2023). In Part E, optional information on energy and water consumption at the site could be provided. Datasets collected focused on one hospital site, which was defined as a specific geographical location where a hospital is operated, assuming that more precise information can be obtained on the basis of the respective scope of services. All data were anonymized, and running dataset numbers were used for identification only.

**Figure 1.**
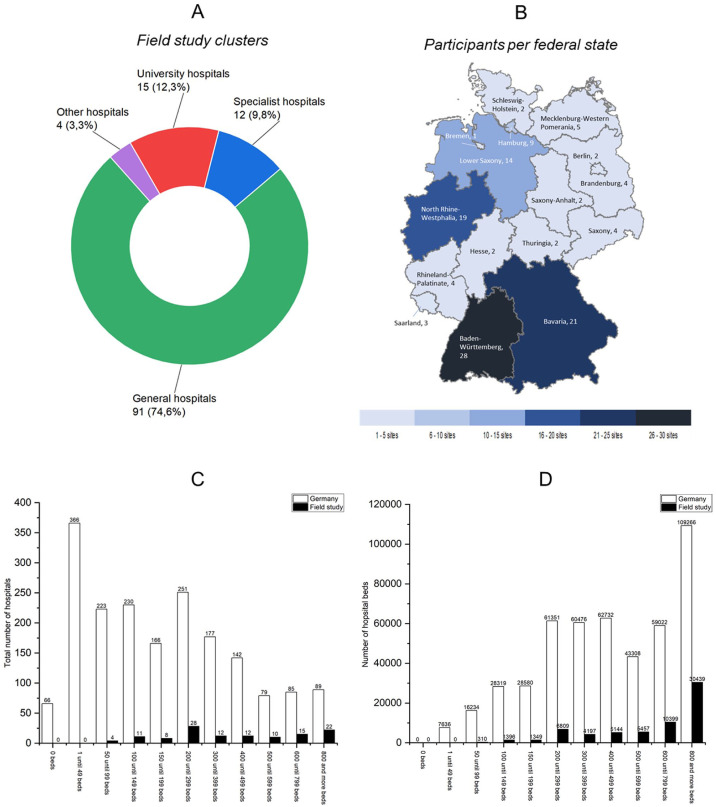
Data structure of field study: A) field study clusters; B) participants per federal state; C) number of hospitals included; D) number of hospital beds included

Data collection and cleaning yielded 122 usable datasets from participating hospital locations from all 16 federal states (Figure 1(b)), and covering four clusters (Figure 1(a)): General hospitals (*n* = 91), university hospitals (*n* = 15), specialist hospitals (*n* = 12) and other hospitals (*n* = 4), which according to the Federal Statistical Office (2023) refers to ‘all hospitals with exclusively psychiatric, psychotherapeutic or psychiatric, psychotherapeutic, and neurological and/or geriatric beds, as well as purely day and night clinics’. Figure 1(c) compares the hospital sizes of the sample with the total number of hospitals in Germany, and Figure 1(d) with regard to the number of beds. The grouping and the number of German hospitals (in total 1874) are taken from the statistical hospital report 2023 on the basic data of German hospitals from the Federal Statistical Office (2024b). However, it should be noted that these statistical reports focus on ‘economic units’, but a hospital as one economic unit may comprise several independently managed locations (sites), specialized clinics or specialist departments (Federal Statistical Office, 2024b). About 14% of all German hospital beds (476,924) were covered by this study (49.88% at university hospitals). Although only 15 datasets were available for the university hospitals, this corresponds to around 44% of the 34 university hospitals in Germany as a whole (Federal Statistical Office, 2024b). Since the Federal Statistical Office reports only combined data for general hospitals and specialized clinics, an exact participation rate for general hospitals – by number of hospitals or beds – cannot be calculated. The two clusters of specialist clinics and other clinics were excluded from the statistical analysis due to the comparatively sparse data available and the different structure of the facilities. For benchmarking at the cluster level, each participant received an individualized dataset including key performance indicators (KPIs) and waste types.

### Waste and structural data correlation

All waste types generated by at least 75% of participants (waste types 18 01 04, 18 01 03*, 20 01 01, 18 01 02, 15 01 01) and all structural parameters reported by at least 75% of participants (beds, inpatient cases, outpatient cases, employees in full-time equivalents (FTE), headcount, physicians in FTE, nurses in FTE, inpatient surgeries, outpatient surgeries, occupancy days, annual turnover) were initially included in the statistical analysis, with three exceptions: The waste codes 20 03 01 (mixed municipal waste) and 18 01 01 (pointed and sharp objects), which together with waste code 18 01 04 constitute the hospital-wide residual waste, were also included in the analysis. Despite the low indication rate of 34.9%, the number of infectious cases (infectious here means ‘contaminated with reportable pathogens according to §§ 6 and 7, also in conjunction with § 15 of the Infection Protection Act (Infektionsschutzgesetz)’ (LAGA, 2021)) was also initially examined.

For correlations, the Pearson correlation requirements were checked for linearity, outliers and scale level. It became apparent that the correlation calculation for the individual waste types would not work due to non-linearity, which can be attributed to two reasons: Firstly, because the waste codes contain a relatively high number of zero entries (e.g. waste type 18 01 02); secondly, because the waste codes are similar in content (e.g. waste type 15 01 01 (paper and cardboard packaging) and waste type 20 01 01 (paper and cardboard), as well as waste type 18 01 04 (waste without infection prevention requirements) and waste type 20 03 01 (mixed municipal waste)) that many study participants use either one or the other, or both to varying degrees, in their waste management for what is essentially the same type of waste.

This resulted in the definition of the three study waste clusters ‘residual waste’ (sum of waste type mass of 18 01 01, 18 01 04 and 20 03 01; 60.6% of the total waste mass), ‘paper and cardboard’ (sum of waste types 15 01 01 and 20 01 01; 11.1% of the total waste mass) and ‘total waste’ (103,432 Mg) for correlation analysis.

**Figure 2.**
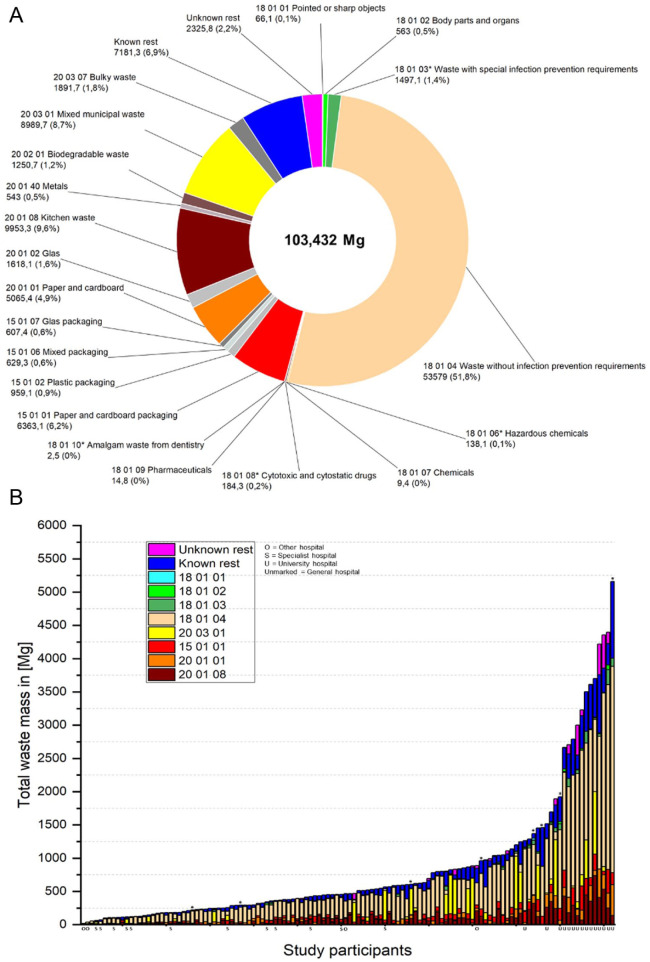
Field study waste data: composition and mass distribution. (a) Composition of the total waste mass documented in the field study in (wt%). (b) Waste composition per hospital site in absolute masses.

The Pearson correlation requirements were tested for all structural parameters and the three study waste clusters. Although meeting linearity requirements, significant outliers in the structural parameters of the outpatient cases at two university hospitals were observed. Consequently, these and the resulting total number of cases in these two clinics were removed from the overall evaluation. Due to data anomalies, the total waste mass reported by the study participants (Figure 2) was excluded from the correlation calculation for eight datasets (marked with * in Figure 2(b)). A metric scale level was determined for all variables.

**Figure 3.**
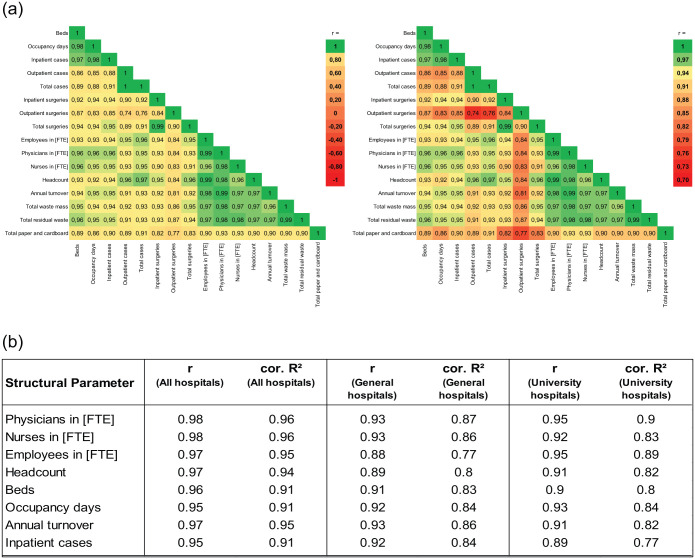
Pearson correlation results. (a) Correlation matrix with two different scale ranges for the Pearson correlation coefficient *r*. (b) Correlation between selected structural parameters and residual waste by hospital cluster.

As the Spearman correlation did not produce any improved results, an overall Pearson correlation matrix (Figure 3(a)) was created, with *r* = ±1 indicating perfect, *r* = 0 no, 0 < |*r*| < 0.3 weak, and |*r*| ⩾ 0.7 strong linear correlations.

In order to examine whether there are significant differences between the two hospital clusters of general hospitals and university hospitals with regard to the correlations between waste and structural parameters, for the eight most strongly correlated structural parameters from the correlation matrix (Figure 3(a)), grouped regressions, in which a separate regression model is estimated for each hospital subgroup, were graphically and mathematically modelled with the study waste cluster residual waste. As the individual types of waste in this cluster (waste types 18 01 01, 18 01 04 and 20 03 01) were specified directly in the data query and these wastes together represent by far the largest proportion of the total waste, the residual waste study cluster was selected for modelling.

### *T*-test for subgroup comparison of KPIs

In order to identify possible differences between the two clusters of general hospitals (hospital cluster A) and university hospitals (B), a two-sample *t*-test was performed on independent samples for each of the eight KPIs calculated for the study waste cluster residual waste, which in turn result from the eight structural parameters that correlate most strongly with residual waste. The normality assumption for the *t*-tests was assessed using *Q*-*Q* plots, which consistently indicated good agreement with normality, supported by the Kolmogorov–Smirnov test (large samples, cluster A) and the Shapiro–Wilk test (small samples, cluster B). While the unpaired *t*-test – and particularly Welch’s *t*-test – is rather robust to moderate deviations from normality (Pagano, 2010; Rasch and Guiard, 2004), in cases where a deviation was detected by the formal tests, the non-parametric Mann–Whitney *U* test was performed to validate the results. If both methods yielded consistent results, the *t*-test was retained. The null hypothesis in each case was that there was no difference in the individual KPIs between the two clusters. The t-test was used five times, and the Welch *t*-test was used three times due to different variances, each of which was tested in advance using the Levene test. All tests were carried out with a confidence level of α = 0.05.

### Mass extrapolation based on field study data

In order to extrapolate the mass of waste group 18 01 from hospitals throughout Germany on the basis of the data from the field study, the previously determined results from the *t*-test were taken into account for the development of a mathematical model. Based on the identified hospital cluster differences between general hospitals and university hospitals, an extrapolation model (1) was created using the number of beds as a reference value:

(1) $$\begin{array}{l} M_{DE,1801}=\left({\bar{m}}_{GH,\;1801}\cdot B_{GH,total}\right)+\left({\bar{m}}_{UH,1801}\cdot B_{UH,\;total}\right)\\ +\left(\left(0.55\cdot \;{\bar{m}}_{GH,\;1801}\right)\cdot B_{OH,total}\right) \end{array}$$

$M_{DE,1801}$ : Total mass of waste group 18 01.

${\bar{m}}_{GH}$ : Average specific waste mass of waste group 18 01 per bed in general hospitals.

$B_{GH,\;total}$ : Total number of hospital beds in general hospitals as well as specialist hospitals.

${\bar{m}}_{UH}$ : Average specific waste mass of waste group 18 01 per bed in university hospitals.

$B_{UH,\;total}$ : Total number of hospital beds in university hospitals.

$B_{OH,total}$ : Total number of hospital beds in other hospitals.

It should be noted that the extrapolation factor for general hospitals also applies to specialist hospitals, which was assumed based on the field study results despite the poor data situation for specialist hospitals. Moreover, bed numbers for specialized hospitals are included in general hospital figures anyway. For other hospitals, a minimization factor of 0.55 was assumed for the extrapolation factor ${\bar{m}}_{GH}$ for general hospitals, which again was based on the field study results using the four datasets from ‘other hospitals’. A prediction model especially for residual waste in general hospitals, based on structural hospital data, can be found in the Supplemental Appendix.

## Results and discussion

### Field study waste data

A total waste mass of 103,432 Mg was documented for 2023 from 122 German hospital locations. Waste type 18 01 04 accounted for the largest mass share at 51.8%, followed by waste type 20 08 01 (kitchen and canteen waste) at 9.6% and mixed municipal waste type 20 03 01 at 8.7% (Figure 2(a)).

Despite differences between WHO and German AVV waste categories, about 9 % of the total waste was hazardous and 91% non-hazardous (assuming 18 01 04 is non-hazardous), which is close to the WHO’s typical range of 10–25% hazardous and 75–90% non-hazardous healthcare waste (WHO, 2014). An arithmetic mean value of 847.8 Mg/site and a median of 465.3 Mg/site were found (Figure 2(b)). The minimum absolute mass was 18.8 Mg, and the maximum at 5159.3 Mg. The waste type 20 01 08 (biodegradable kitchen waste) result should be treated with caution, as some participants indicated incomplete collection both of the wastes and the waste information in their facilities. For other types of waste, such as 15 01 07 (glass packaging), 20 01 02 (glass) but also 15 01 02 (plastic packaging) and 15 01 06 (mixed packaging), no specific mass data could be recorded in some cases as their collection and logistics were outsourced to third parties. For 8 of the 122 respondents, the total waste mass indicated was below the sum of the individual fractions (marked with an asterisk in Figure 2(b)). To improve data quality, these datasets were excluded from all calculations relating to the total waste mass.

From the remaining data, it was possible to calculate the total waste per bed and day as 4.23 kg/bed/day for the entire field of participants, 3.57 kg/bed/day for the general hospitals and 5.58 kg/bed/day for the university hospitals. Overall, a rate of approximately 0.38 kg/bed/day of hazardous waste and 3.95 kg/bed/day of non-hazardous waste was calculated. Compared with selected international values, the value calculated for the entire field of participants ranks behind the USA (8.4 kg/bed/day; Mewaldt et al., 2023) and ahead of Iran (4.0 kg/bed/day; Khazaee et al., 2015). For the KPI total waste per case, the overall result is 8.33 kg/case, 9.9 kg/case for the general hospitals and 6.93 kg/case for the university hospitals. The values for the total waste mass per FTE are 565.28 kg/FTE/year or 1.55 kg/FTE/day (all participants), 674.41 kg/FTE/year (general hospitals) and 461.18 kg/FTE/year (university hospitals). Chapter 3.4 ‘Data comparison between official waste statistics and benchmark study’ presents a model for extrapolating the waste data from the field study to the German hospitals landscape as a whole.

All German values for total waste intensity in kg/bed/day are above the global median of 1.2 kg/bed/day, regardless of the type of hospital, and are among the highest in international comparison (Ansari et al., 2019; Singh et al., 2022), while the German rate of hazardous waste is only slightly below the global median of 0.46 kg/bed/day (Mol et al., 2022). This high total rate reflects Germany’s status as a wealthy industrialized nation, consistent with studies linking higher GDP and healthcare spending to increased hospital waste (Minoglou et al., 2017; Windfeld and Brooks, 2015). The differences between hospital types are largely explained by staffing structures, as further detailed in the following chapter. But surgical activity also contributes: university hospitals perform 27.8 operations per bed annually versus 20.5 in general hospitals, supporting literature identifying surgeries as key waste drivers (Cesaro and Belgiorno, 2017; Friedericy et al., 2022; MacNeill et al., 2017; Wyssusek et al., 2019). For kg/case no comparative data were found, while kg/FTE/day in Germany is more than twice the value reported for Palestine (0.67 kg/FTE/day; Eleyan et al., 2013), again reflecting the link between higher GDP/capita and waste generation.

### Key performance indicator identification

The results for all general and university hospitals together (*N* = 106) show that there are exclusively strong Pearson correlations between the 13 structural parameters and the 3 analysed hospital waste study clusters (Figure 3(a)).

There are only positive correlations between the variables. The correlations of structural parameters with the total waste mass and the residual waste, for which 5 of 13 correlation coefficients are the same, are always stronger than those with the paper waste.

The study waste clusters ‘total waste’ and ‘residual waste’ have a very high correlation coefficient of 0.99, suggesting that these two variables can be used interchangeably, especially if the large mass share of this waste group of 61.4% of the total waste mass is also taken into account. Moreover, the structural parameter full-time physicians (FTE) shows the strongest correlations with all three study waste clusters. While a correlation coefficient of 0.98 was also calculated for the correlation with full-time nurses (FTE) for residual waste, this is 0.97 for total waste and 0.93 for paper waste. For total waste and residual waste together, the following variables are employees in FTE (0.97), headcount (0.97) and total annual revenue (0.96 and 0.97, respectively), which also show strong linear correlations, as well as beds (0.95 and 0.96, respectively), occupancy days and inpatient cases (both 0.94 and 0.95, respectively). The further case-associated and surgery-associated correlation coefficients are slightly lower overall. The weakest correlations are found in the structural parameter outpatient surgeries.

In addition, the results of the grouped regressions using the example of residual waste show that both the calculated Pearson correlation coefficients and the corresponding *R*^2^ values of the linear regressions (Figure 3(b)) for the total population (‘all hospitals’) are always higher than for the two clusters of university hospitals and general hospitals. This could be due to the fact that the overall dataset contains a higher variance of data points than the individual clusters, which can reinforce the linear relationships of the overall dataset. Minor outliers are also smoothed out by aggregation. Simpson’s Paradox, which describes the amplification of common trends through aggregation, could also be responsible. Although there are only 15 data points in the university hospitals cluster, the lowest value for the correlation coefficient is 0.89 and for the *R*^2^ value 0.77 (both for inpatient cases). In addition to strong correlations, this also indicates good data quality.

The results of the correlation analysis show that there are other scientifically sound alternatives to the widely applied unit ‘kilogram per bed per day’ (kg/bed/day), which dominates the literature on hospital waste generation (Ansari et al., 2019; Mol et al., 2022; Singh et al., 2022). Other quantification units such as kg/patient/day (Cheng et al., 2010; Eleyan et al., 2013; Gao et al., 2018; Sawalem et al., 2009) or kg/employee/day (Eleyan et al., 2013) have been used much less frequently to date, but they should definitely be considered as suitable indicators for quantifying waste generation in hospital solid waste management. But suitable KPIs could also be calculated from other structural parameters such as the total annual turnover, the occupancy days and the two employee-related structural parameters physicians in FTE and nurses in FTE. Interestingly, the number of employees consistently showed the strongest correlations with waste volumes, highlighting a potentially fundamental link between staff numbers and waste generation. The reason for the predominant use of the parameter waste/bed/day to date is likely that the number of beds is a ubiquitous and tangible parameter in hospital practice, which was also reflected in the 100% response rate for this parameter in this field study. However, the consistently strong correlations with other structural factors indicate that the current use of KPIs in the literature risks oversimplifying the complex causes of waste generation in hospitals. The broader application of alternative indicators – particularly those related to staff – would allow for a more nuanced benchmarking and could potentially provide further insights for optimizing waste management strategies.

In addition to the correlation analysis, the graphically displayed grouped linear regressions of the examined KPIs in the two relevant hospital clusters (see Figure 5) also provide important insights, particularly regarding the impact of differences in staffing structures across different types of hospitals. The analysis of the structural parameters headcount and employees in FTE show great similarities in the course of their regression lines (Figure 5). Both diagrams show the flatter regression line for university hospitals and the steep regression line for general hospitals. This leads to a kind of downward bend.

**Figure 4.**
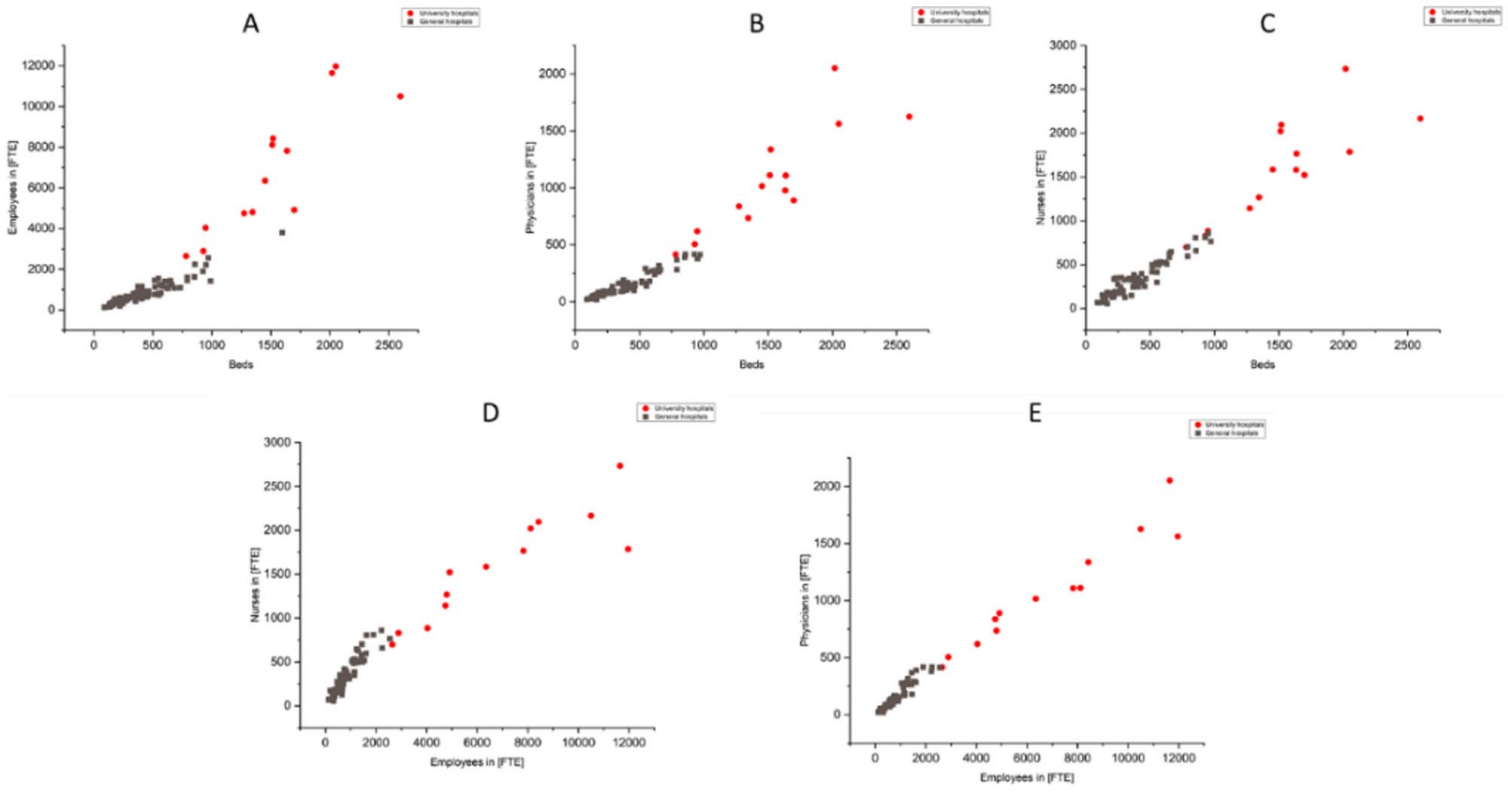
Graphical representation of relevant relationships of structural parameters: A) employees in relation to beds; B) physicians in relation to beds; C) nurses in relation to beds; D) nurses in relation to employees; E) physicians in relation to employees.

Individual diagrams (a–e) in Figure 4 show that university hospitals have more employees in FTE per hospital bed (a), including noticeably more physicians (b). In the case of nursing staff per bed, this is slightly less pronounced, so that there is more of a linear trend across the two hospital clusters (c). But it should be emphasized that the share of these nursing staff in total staffing is significantly lower in the university hospitals. This can also be seen in the pronounced kink (d). In addition, the proportion of physicians, which is significantly higher per bed at the university hospitals, is expressed as a proportion of the total workforce across both clusters in a linear approximation (e).

It can be said that the courses of the linear regression lines in the two clusters of university hospitals and general hospitals are largely similar with regard to the structural parameters that relate to the overall organization (e.g. number of employees, employees in FTEs and total annual turnover). There are also comparable trends in the regression curves in the two clusters examined for the structural parameters that relate exclusively to direct patient care (number of beds, occupancy days, inpatient cases).

These observations and effects indicate that university hospitals have a different personnel structure compared to general hospitals, with a major impact on waste generation. For example, the proportion of employees who are involved in direct, waste-intensive patient care must be significantly higher in general hospitals (share of physicians and nurses in the total number of FTE: 40% in university hospitals and 60% in general hospitals). At university hospitals, on the other hand, the proportion of employees in research and teaching, for example, who are less waste-intensive, is noticeably higher.

**Figure 5.**
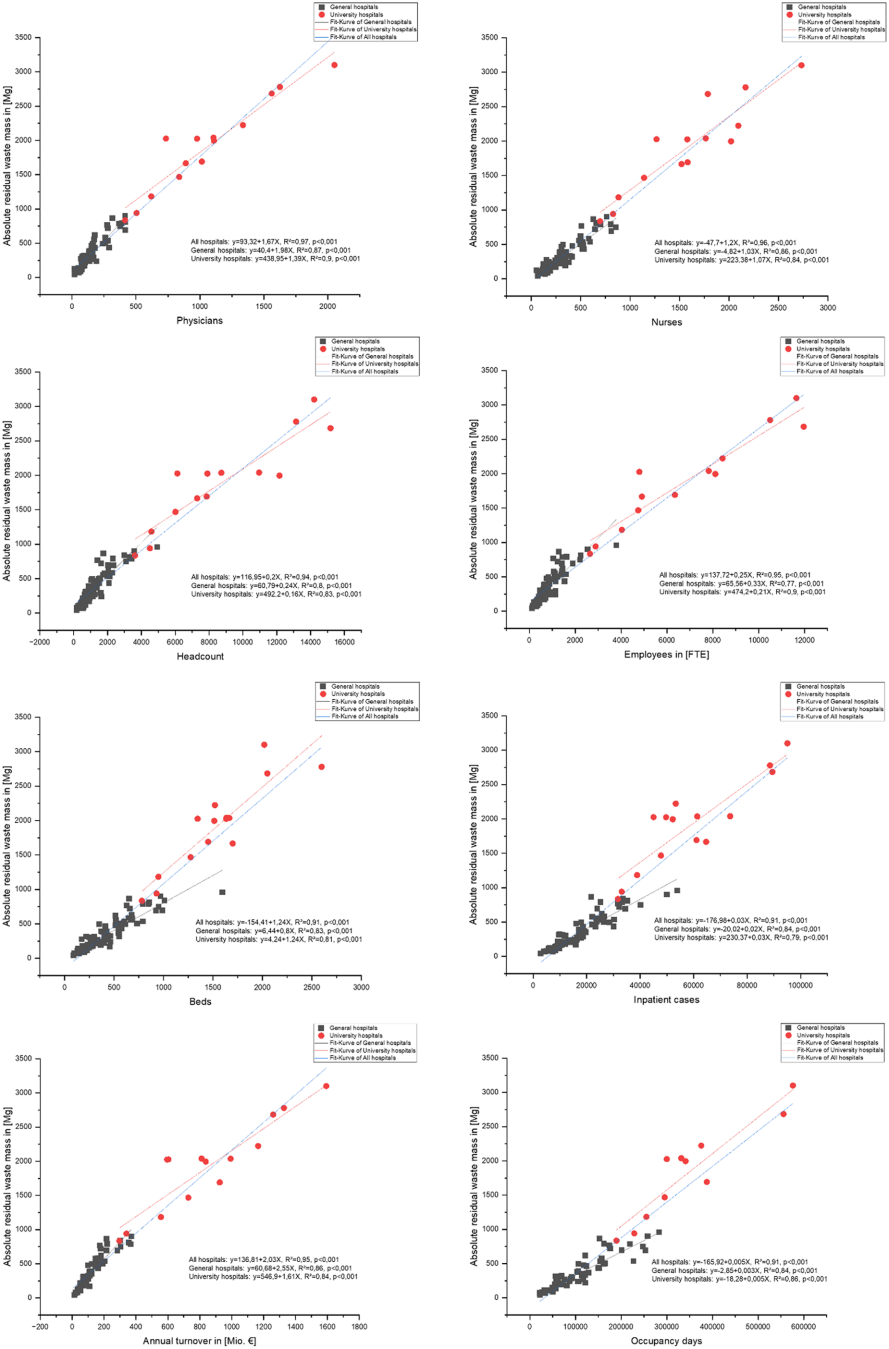
Linear regressions.

These results indicate that hospital waste generation primarily depends on staffing structure, particularly the proportion of staff directly involved in patient care. This is consistent with the high correlations shown in Figure 3. However, it should also be noted that other factors may also contribute to differences in waste generation: For instance, university hospitals generate less waste per euro of revenue than general hospitals (Figure 5). One reason for this could be that university hospitals generate a higher proportion of their turnover from non-patient-related sources (e.g. research and teaching), which produce less waste. Although no studies analyse explicitly differences between hospital types as done here, the literature consistently shows that facility type affects waste generation (Bendjoudi et al., 2009; Cheng et al., 2010; Sawalem et al., 2009; Taghipour and Mosaferi, 2009; Wiafe et al., 2015). Other factors include waste management methods, the use of reusable products, specialization level, patient volume and patient socioeconomic background (Taghipour and Mosaferi, 2009), as well as the location and the equipment utilization (Sawalem et al., 2009), surgical activity (Cesaro and Belgiorno, 2017), the inpatient-to-outpatient ratio and case mix. Overall, while staffing – especially the proportion of direct care staff – is the main factor explaining differences in waste generation between university and general hospitals, waste also results from a complex interplay of organizational, clinical and contextual determinants (Taghipour and Mosaferi, 2009). Their relative contributions remain unclear and should be quantified in future research to guide sustainable hospital waste management.

### Hospital cluster KPIs

**Figure 6.**
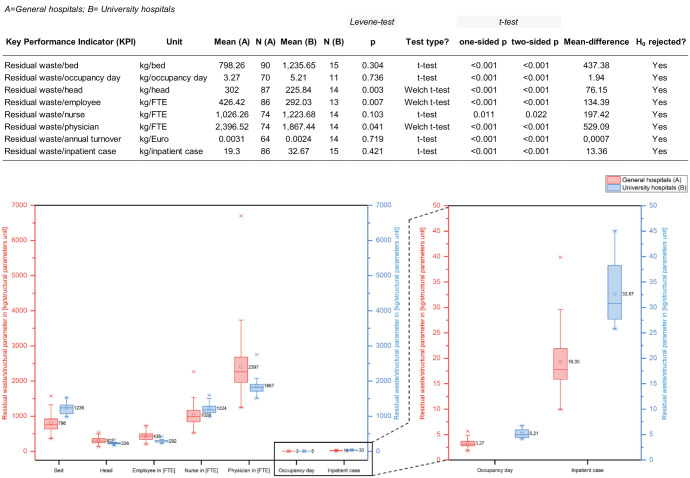
*T*-test results (residual waste/turnover not shown in boxplots due to different scale range).

The results in Figure 6 show that in all *t*-tests, the null hypothesis was rejected because the significance level of α = 0.05 was not reached. Instead, the alternative hypothesis that there are significant differences between the two clusters of general hospitals (A) and university hospitals (B) was accepted. For the KPIs residual waste/physician and residual waste/turnover, the Shapiro–Wilk test indicated a deviation from a normal distribution for the university hospital data, even though the *Q*-*Q* plots appeared to show a normal distribution. However, the Mann–Whitney *U* test yielded the same result as the Welch test and the *t*-test, so these results were retained, as described in the Materials and methods chapter.

The different sample sizes in the two clusters (Figure 6) are due to the limited number of university hospitals and are considered uncritical. The fluctuating values for the sample size are due to data gaps in the indication of the respective structural parameters.

University hospitals show significantly higher values for waste/bed, waste/nurse, waste/occupancy day and waste/inpatient case, while the general hospitals exhibit higher waste/head, waste/employee and waste/physician values. The respective higher values of the two clusters are expressed analogously in the previous chapter by the same higher values for the slopes of the corresponding regression line (Figure 5). These differences reflect the distinct staffing structures of the two hospital clusters, which make direct KPI comparisons difficult and highlight the need for cluster-based benchmarking.

Comparable findings are reported in the literature: Bendjoudi et al. (2009) observed 255.5–401.5 kg/bed of infectious waste in Algerian university hospitals versus 73–255.5 kg/bed in general hospitals, and Taghipour and Mosaferi (2009) reported 1251.95 kg/bed of total waste in Iranian university hospitals compared to 1111.8 kg/bed in governmental hospitals. Although largely limited to waste/bed KPIs, these studies confirm the strong influence of staffing structures and other potential influencing factors, which were identified in the regression analyses and have already been discussed in detail (Figure 5).

### Data comparison between official waste statistics and benchmark study

**Figure 7.**
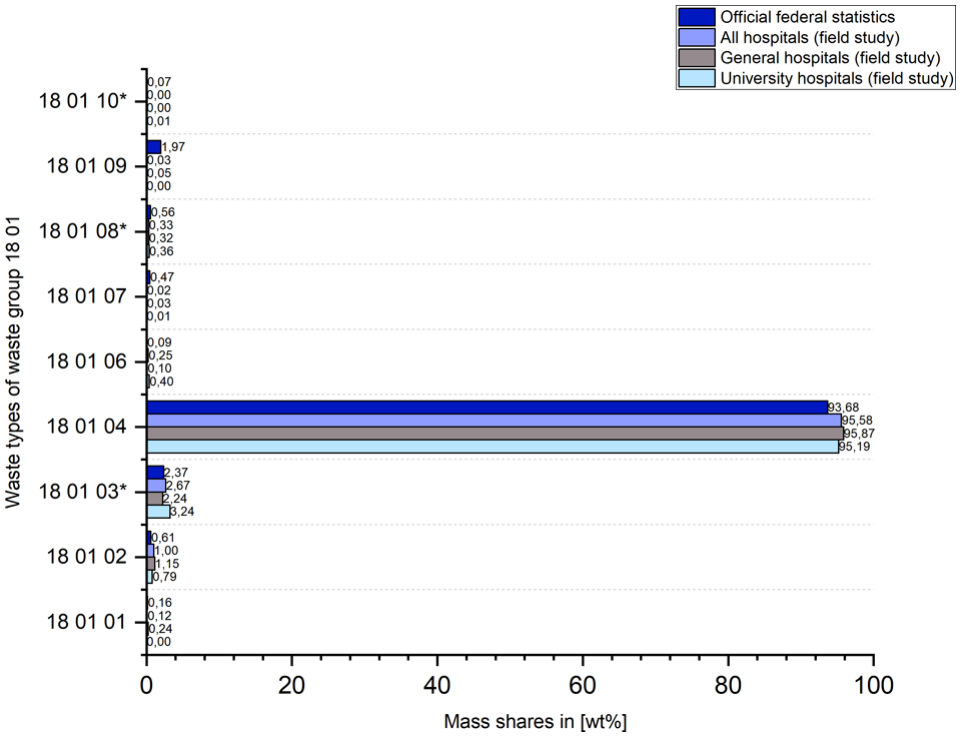
Comparison of the mass shares of individual waste types within waste group 18 01 between official statistics (Federal Statistical Office, 2022) and the field study.

When comparing the mass shares of the nine waste types within waste group 18 01 (i.e. waste attributable to human health care) between all hospital sites of the field study (*N* = 122) as well as the two previously examined hospital clusters university hospitals and general hospitals and the official waste statistics available at federal level (Federal Statistical Office, 2022), little deviation is observed (Figure 7).

In order to extrapolate the total mass of waste group 18 01 generated in hospitals for Germany based on the data from the field study in order to establish a comparison with the values from the federal statistics (Federal Statistical Office, 2022), it is not sufficient to implement this using the number of study participants or hospitals in Germany as an extrapolation factor. On the one hand, this is because the survey units (local hospital site in the field study and economic unit in the statistical hospital report) are not congruent. On the other hand, the data structure of the field study participants shown in Figure 1 would not be taken into account. Figure 1 clearly shows that mainly large hospitals with many beds, which logically also produce more waste, took part in the study. Smaller hospitals, on the other hand, which make up a significant proportion of the total number of hospitals in terms of numbers but produce less waste, are hardly represented or not represented at all (e.g. in the largest size category by far, 0–49 beds). Overall, the field study covers almost 14% of German hospital beds, while only about 6.5% of the total number of hospitals are covered (based on a 1:1 conversion of local hospital locations and economic units).

Since the number of planned beds was stated by all study participants and this variable is also shown for Germany in the statistical hospital report (Federal Statistical Office, 2024b) and cluster differences for the waste/bed parameter could already be identified in this study, which can be directly included in the extrapolation model, the number of beds was selected as a suitable extrapolation variable.

Based on the annual masses of waste group 18 01 documented in the field study (general hospitals: 25,820 Mg; university hospitals: 28,120 Mg) and the corresponding bed counts (general hospitals: 38,607; university hospitals: 23,068), the two necessary extrapolation factors 668.8 kg/bed (general hospitals) and 1219 kg/bed (university hospitals) are calculated. When these are offset against the bed counts from the statistical hospital report (general hospitals: 382,545 beds; university hospitals: 46,246 beds; other hospitals: 48,133 beds) according to formula (1), the total waste mass for waste group 18 01 from German hospitals for 2023 is calculated by 329,925.21 Mg.

While the total waste mass of waste group 18 01 according to official statistics for Germany is 425,400 Mg (Federal Statistical Office, 2022), the value of the present study is 95,474.79 Mg lower than the federal statistics. It may be suggested that the share of waste from sources other than hospitals must be significant. Since the data source for the federal statistics is waste disposal companies and not the facilities themselves, no further information is available on the actual mass shares of the various healthcare stakeholders (e.g. laboratories, nursing homes, medical practices), thus opening up scope for further research.

Using the same formula (1) exclusively for the extrapolation of the nationwide waste mass of type 18 01 03* from hospitals on the basis of the field study data, a value of 7947 Mg is obtained, which, compared with the corresponding value from the federal statistics (10,100 Mg/a), indicates around 21.3% of sources other than hospitals.

Considering that the total waste mass of waste group 18 01 (56,054 Mg) accounts for around 54.2% of the total waste mass documented in the field study (103,432 Mg) across all participating hospitals, the extrapolated value above results in a roughly estimated total annual waste mass of all waste types of around 608,718.1 Mg from German hospitals.

In summary, it can be stated that there are great similarities between the federal statistics and the field study regarding the mass distribution of the individual waste types in waste group 18 01. However, it must be critically noted that the field study cannot provide any information on the composition of the two largest mass shares, 18 01 03 and 18 01 04, within the hospital-specific waste group in terms of content, material and object. Furthermore, using an extrapolation model developed based on the field study, it was determined that a significant proportion (22.44%) of the waste mass reported in the federal statistics must originate from sources other than hospitals.

## Conclusion

The correlation analysis shows that waste/bed/day is an inadequate single KPI for quantifying hospital waste, while personnel-related metrics such as waste/FTE/day are more informative and should be given greater consideration in future. Statistical tests confirm significant differences between university and general hospitals, primarily attributable to differing staffing structures: General hospitals have a higher proportion of staff directly involved in patient care, while university hospitals employ a larger proportion of staff in research and teaching. Furthermore, extrapolating the benchmark study data suggests that German hospitals generate approximately 330,000 metric tonnes of waste of category 18 01, while about 22% of the quantities recorded in official statistics originate from other healthcare facilities. These findings underscore the need for future waste analyses to incorporate personnel-related KPIs, for KPI comparisons to be conducted on a cluster-specific basis, and for sustainable waste management to consider both staffing structures and all relevant stakeholders in the healthcare sector.

## Supplemental Material

sj-docx-1-wmr-10.1177_0734242X251405987 – Supplemental material for Healthcare waste in German hospitals: A nationwide benchmark study Supplemental material, sj-docx-1-wmr-10.1177_0734242X251405987 for Healthcare waste in German hospitals: A nationwide benchmark study by Anton Vielsack, Viola Galler, Anne-Kathrin Cassier-Woidasky, Franziska Zecha and Joerg Woidasky in Waste Management & Research

## References

[bibr1-0734242X251405987] Ansari M Ehrampoush MH Farzadkia M , et al. (2019) Dynamic assessment of economic and environmental performance index and generation, composition, environmental and human health risks of hospital solid waste in developing countries; A state of the art of review. Environment International 132: 105073. 31421384 10.1016/j.envint.2019.105073

[bibr2-0734242X251405987] Bendjoudi Z Taleb F Abdelmalek F , et al. (2009) Healthcare waste management in Algeria and Mostaganem department. Waste Management 29: 1383–1387. 19091540 10.1016/j.wasman.2008.10.008

[bibr3-0734242X251405987] Cesaro A Belgiorno V (2017) Sustainability of medical waste management in different sized health care facilities. Waste and Biomass Valorization 8: 1819–1827.

[bibr4-0734242X251405987] Cheng YW Li K-C Sung FC (2010) Medical waste generation in selected clinical facilities in Taiwan. Waste Management (New York, N.Y.) 30: 1690–1695. 20427173 10.1016/j.wasman.2010.04.006

[bibr5-0734242X251405987] Diaz LF Eggerth LL Enkhtsetseg S , et al. (2008) Characteristics of healthcare wastes. Waste Management (New York, N.Y.) 28: 1219–1226. 17651963 10.1016/j.wasman.2007.04.010

[bibr6-0734242X251405987] Eleyan D Al-Khatib IA Garfield J (2013) System dynamics model for hospital waste characterization and generation in developing countries. Waste Management & Research 31: 986–995. 23743573 10.1177/0734242X13490981

[bibr7-0734242X251405987] Federal Statistical Office (2008) Klassifikation der Wirtschaftszweige 2008 (WZ 2008). Available at: https://www.destatis.de/DE/Methoden/Klassifikationen/Gueter-Wirtschaftsklassifikationen/klassifikation-wz-2008.html (accessed 29. December 2025).

[bibr8-0734242X251405987] Federal Statistical Office (2024a) Qualitätsbericht - Erhebung der Abfallerzeugung 2022. Available at: https://www.destatis.de/DE/Methoden/Qualitaet/Qualitaetsberichte/Umwelt/einfuehrung.html (accessed 29 December 2025).

[bibr9-0734242X251405987] Federal Statistical Office (2022) Datenabruf: Abfallentsorgung: Deutschland, Jahre (2022), Abfallarten. Available at: https://www-genesis.destatis.de/genesis/online?operation=ergebnistabelleUmfang&levelindex=2&levelid=1702652803980&downloadname=32111-0002#abreadcrumb (accessed 30 August 2024).

[bibr10-0734242X251405987] Federal Statistical Office (2023) Basic data on hospitals (2022) - Grunddaten der Krankenhäuser (2022). Available at: https://www.statistischebibliothek.de/mir/receive/DEHeft_mods_00153799 (accessed 29 December 2025).

[bibr11-0734242X251405987] Federal Statistical Office (2024b) Statistical report - Basic hospital data: Statistischer Bericht - Grunddaten der Krankenhäuser. Available at: https://www.destatis.de/DE/Themen/Gesellschaft-Umwelt/Gesundheit/Krankenhaeuser/Publikationen/_publikationen-innen-grunddaten-krankenhaus.html (accessed 23 April 2025).

[bibr12-0734242X251405987] Feld M Riedel M Schmidt J , et al. (2023) Kreislaufwirtschaftliche Ansätze für das Gesundheitswesen: Circular economy approaches for the health care sector. Müll und Abfall 55: 601–607.

[bibr13-0734242X251405987] Friedericy HJ van Egmond CW Vogtländer JG , et al. (2022) Reducing the environmental impact of sterilization packaging for surgical instruments in the operating room: A comparative life cycle assessment of disposable versus reusable systems. Sustainability 14: 430.

[bibr14-0734242X251405987] Gao Q Shi Y Di Mo , et al. (2018) Medical waste management in three areas of rural China. PLoS One 13: e0200889. 10.1371/journal.pone.0200889 PMC6054418 30028841

[bibr15-0734242X251405987] Hunger S Kemter-Esser K Luginsland M , et al. (2023) White Paper “ReMed”: Mit werkstofflichem Recycling zu einer nachhaltigen Medizintechnik - Herausforderungen und Lösungsansätze für die Verarbeitung von Klinikabfällen. Available at: https://publica-rest.fraunhofer.de/server/api/core/bitstreams/eed25dd0-89e8-4519-98fd-ed862603fbab/content (accessed 29 December 2025).

[bibr16-0734242X251405987] Karliner J Slotterback S Boyd R , et al. (2019) Health care’s climate footprint: The health sector contribution and opportunities for action. In: 16th World Congress on Public Health, 12–16 October 2020.

[bibr17-0734242X251405987] Khazaee Hamidian Taheri , et al. (2015) Assessment of medical waste management in Karaj Hospitals, Iran. International Research Journal of Applied and Basic Sciences 9: 1750–1757.

[bibr18-0734242X251405987] LAGA (2021) Vollzugshilfe zur Entsorgung von Abfällen aus Einrichtungen des Gesundheitsdienstes. Available at: https://www.laga-online.de/documents/laga-m-18_stand_2021-06-23_1626849905.pdf (accessed 29 December 2025).

[bibr19-0734242X251405987] MacNeill AJ Lillywhite R Brown CJ (2017) The impact of surgery on global climate: A carbon footprinting study of operating theatres in three health systems. The Lancet. Planetary Health 1: e381–e388. 10.1016/S2542-5196(17)30162-6 29851650

[bibr20-0734242X251405987] Mewaldt C Armand W Slutzman J , et al. (2023) The plastic pandemic: Quantification of waste on an inpatient medicine unit. The Journal of Climate Change and Health 11: 100230.

[bibr21-0734242X251405987] Minoglou M Gerassimidou S Komilis D (2017) Healthcare waste generation worldwide and its dependence on socio-economic and environmental factors. Sustainability 9: 220.

[bibr22-0734242X251405987] Mol MPG Zolnikov TR Neves AC , et al. (2022) Healthcare waste generation in hospitals per continent: A systematic review. Environmental Science and Pollution Research International 29: 42466–42475. 35364785 10.1007/s11356-022-19995-1

[bibr23-0734242X251405987] Pagano RR (2010) Understanding Statistics in the Behavioral Sciences. Belmont, CA: Wadsworth Publishing Co Inc.

[bibr24-0734242X251405987] Rasch D Guiard V (2004) The robustness of parametric statistical methods. Psychology Science 46: 175–208.

[bibr25-0734242X251405987] Sawalem M Selic E Herbell J-D (2009) Hospital waste management in Libya: A case study. Waste Management (New York, N.Y.) 29: 1370–1375. 19036572 10.1016/j.wasman.2008.08.028

[bibr26-0734242X251405987] Singh N Ogunseitan OA Tang Y (2022) Medical waste: Current challenges and future opportunities for sustainable management. Critical Reviews in Environmental Science and Technology 52: 2000–2022.

[bibr27-0734242X251405987] Slutzman JE Bockius H Gordon IO , et al. (2023) Waste audits in healthcare: A systematic review and description of best practices. Waste Management & Research 41: 3–17. 35652693 10.1177/0734242X221101531 PMC9925917

[bibr28-0734242X251405987] Taghipour H Mosaferi M (2009) Characterization of medical waste from hospitals in Tabriz, Iran. The Science of the Total Environment 407: 1527–1535. 19108872 10.1016/j.scitotenv.2008.11.032

[bibr29-0734242X251405987] WHO (2014) Safe Management of Wastes from Health-care Activities: A Practical Guide. Geneva: World Health Organization.

[bibr30-0734242X251405987] WHO (2022) Global Analysis of Healthcare Waste in the Context of COVID-19: Status, Impacts and Recommendations. Geneva: World Health Organization.

[bibr31-0734242X251405987] Wiafe S Nooni I Nlasia MS , et al. (2015) Assessing clinical solid waste management strategies in Sunyani Municipality, Ghana – Evidence from three healthcare facilities. International Journal of Environment and Pollution Research 3: 32–52.

[bibr32-0734242X251405987] Windfeld ES Brooks MS-L (2015) Medical waste management - A review. Journal of Environmental Management 163: 98–108. 26301686 10.1016/j.jenvman.2015.08.013

[bibr33-0734242X251405987] Wyssusek KH Keys MT van Zundert AAJ (2019) Operating room greening initiatives - the old, the new, and the way forward: A narrative review. Waste Management & Research 37: 3–19. 30132405 10.1177/0734242X18793937

