# Supplementary material for: Healthcare waste in German hospitals: A nationwide benchmark study
Source: Waste Manag Res. 2026 Jan 13;44(6):808–19. doi: 10.1177/0734242X251405987 (PMC13191062; doi:10.1177/0734242X251405987)
Supplement: sj-docx-1-wmr-10.1177_0734242X251405987 – Supplemental material for Healthcare waste in German hospitals: A nationwide benchmark study [file sj-docx-1-wmr-10.1177_0734242X251405987.docx]

***Supplementary Material - Appendix***

**Methodology: Statistical Prediction Model of Waste Mass Using Hospital Structural Data**

The subgroup of general hospitals was used as an example to create a prediction model of the annual residual waste mass, as most of the data was available here. In a first step, the four frequently stated structural parameters number of beds (n=91), number of inpatient cases (n=88), number of employees in full-time equivalents (n=87) and the number of heads (n=88) of the 91 general hospitals were defined as variables representative of the hospital size for further modeling.

Subsequently a principal component analysis (PCA) was carried out to eliminate the problem of multicollinearity. The determined main component “hospital index” was then calculated with the help of formula (2) for each of the general hospitals using the calculated component score coefficients from the component score coefficient matrix and the previously standardized values of the structural parameters. For PCA, all variables were standardized in order to bring them to the same scale.

$$\boldsymbol{PC\_1=}\boldsymbol{b}_{\boldsymbol{1}}\boldsymbol{\cdot}\boldsymbol{z}_{\boldsymbol{1}}\boldsymbol{+}\boldsymbol{b}_{\boldsymbol{2}}\boldsymbol{\cdot}\boldsymbol{z}_{\boldsymbol{2}}\boldsymbol{+}\boldsymbol{b}_{\boldsymbol{3}}\boldsymbol{\cdot}\boldsymbol{z}_{\boldsymbol{3}}\boldsymbol{+}\boldsymbol{b}_{\boldsymbol{4}}\boldsymbol{\cdot}\boldsymbol{z}_{\boldsymbol{4}} (2)$$

$b_{1-4}$ : Component score coefficients for each variable

$z_{1-4}$ : Standardized variables

$PC\_1$ : Principal component “hospital index”

The individual main components were then transferred together with the corresponding annual residual waste masses in [Mg] of the hospitals into a linear regression, the result term of which contains the following elements and can therefore be used to predict the annual residual waste mass for general hospitals with the four known structural parameters:

$$\boldsymbol{y}\boldsymbol{=}\boldsymbol{\beta}_{\boldsymbol{0}}\boldsymbol{+}\boldsymbol{\beta}_{\boldsymbol{1}}\boldsymbol{\cdot PC\_1}\boldsymbol{+\varepsilon}(3)$$

$y$ : Annual residual waste mass – dependent variable (target figure)

$\beta_{0}$ : Axis section – intercept

$\beta_{1}$ : Regression coefficient – gradient

$PC\_1$ : Principal component “hospital index” – independent variable

$\varepsilon$ : Error term

Formula (4) can be used to standardize the four variables or structural parameters as a basic prerequisite for predicting the residual waste mass or calculating the unknown main component. The mean values and standard deviations to be used in general were calculated from the data set of the field study in the case of the model considered here.

$$\boldsymbol{z}_{\boldsymbol{1-4}}\boldsymbol{=}\frac{\boldsymbol{x}_{\boldsymbol{1-4}}\boldsymbol{-}\boldsymbol{\mu}_{\boldsymbol{1-4 (all)}}}{\boldsymbol{\sigma}_{\boldsymbol{1-4 (all)}}} (4)$$

$z_{1-4}$ : Standardized variable (structural parameter) from one hospital

$x_{1-4}$: Known original value of variable (structural parameter) from one hospital

$\mu_{1-4 (all)}$ : Average value of the distribution from the field study data

$\sigma_{1-4 (all)}$ : Standard deviation of the distribution from the field study data

### Results: Prediction of Residual Waste Mass of General Hospitals

In a first calculation step, the PCA of the four structural parameters examined showed that the Kaiser-Meyer-Olkin criterion was 0.858 and the Bartlett test was highly significant (p < 0.001), indicating a sufficiently high correlation between the items. Only factors with eigenvalues ≥ 1 were considered. A review of the Kaiser-Guttmann criterion and the Scree plot led to the extraction of a factor that explained a total variance of 95.4. A rotation was not performed due to the reduction to only one factor.

The principal component “hospital index” of the prediction model was calculated for each of the general hospitals using the four calculated component score coefficients for each structural parameter (employees in full-time equivalents: 0.259, headcount: 0.257, number of inpatient cases: 0.255, number of beds: 0.254) and the standardized values of the structural parameters themselves. In contrast, the factor loadings representing the correlation between the component and each variable calculated were 0.987 for the number of full-time equivalent employees, 0.98 for the headcount, 0.972 for the number of inpatient cases and 0.969 for the number of beds.

The subsequent linear regression with the respective annual residual waste masses in [Mg] of the general hospitals yielded 334.1 for $\beta_{0}$ and 209.5 for $\beta_{1}$. The standard error of the regression coefficient is 9.914. The coefficient of determination R² is 0.842, while the p-value is < 0.001, which indicates statistical significance. This results in the prediction model given in Formula 5:

$$\boldsymbol{y}\boldsymbol{=334.1+209.5\cdot PC\_1}(5)$$

$$\boldsymbol{y=334.1+54.2605*}\boldsymbol{z}_{\boldsymbol{1}}\boldsymbol{+53.8415\cdot}\boldsymbol{z}_{\boldsymbol{2}}\boldsymbol{+53.4225\cdot}\boldsymbol{z}_{\boldsymbol{3}}\boldsymbol{+53.213\cdot}\boldsymbol{z}_{\boldsymbol{4}}$$

$z_{1-4}$ : Standardized variables

By calculating the independent variable $PC\_1$ with the four calculated component score coefficients using formula (2), the annual residual waste mass $y$ in [Mg] for a general hospital can be predicted. The average value of the distribution from the field study data for pre-standardization as in formula (4) is 416.57 for the number of beds, 812 for the full-time equivalents, 1,146.02 for the headcount and 16,818.10 for the inpatient cases. For the standard deviation, which is also necessary, the corresponding values are 257.98 for the number of beds, 597.44 for the number of full-time equivalent employees, 844.91 for the number of heads and 9,903.02 for the number of inpatient cases.
